# Supplementary material for: Host-genotype dependent gut microbiota drives zooplankton tolerance to toxic cyanobacteria
Source: Nat Commun. 2017 Nov 20;8:1608. doi: 10.1038/s41467-017-01714-x (PMC5694789; doi:10.1038/s41467-017-01714-x)
Supplement: Supplementary file 1 — Supplementary Information [file 41467_2017_1714_MOESM1_ESM.pdf]

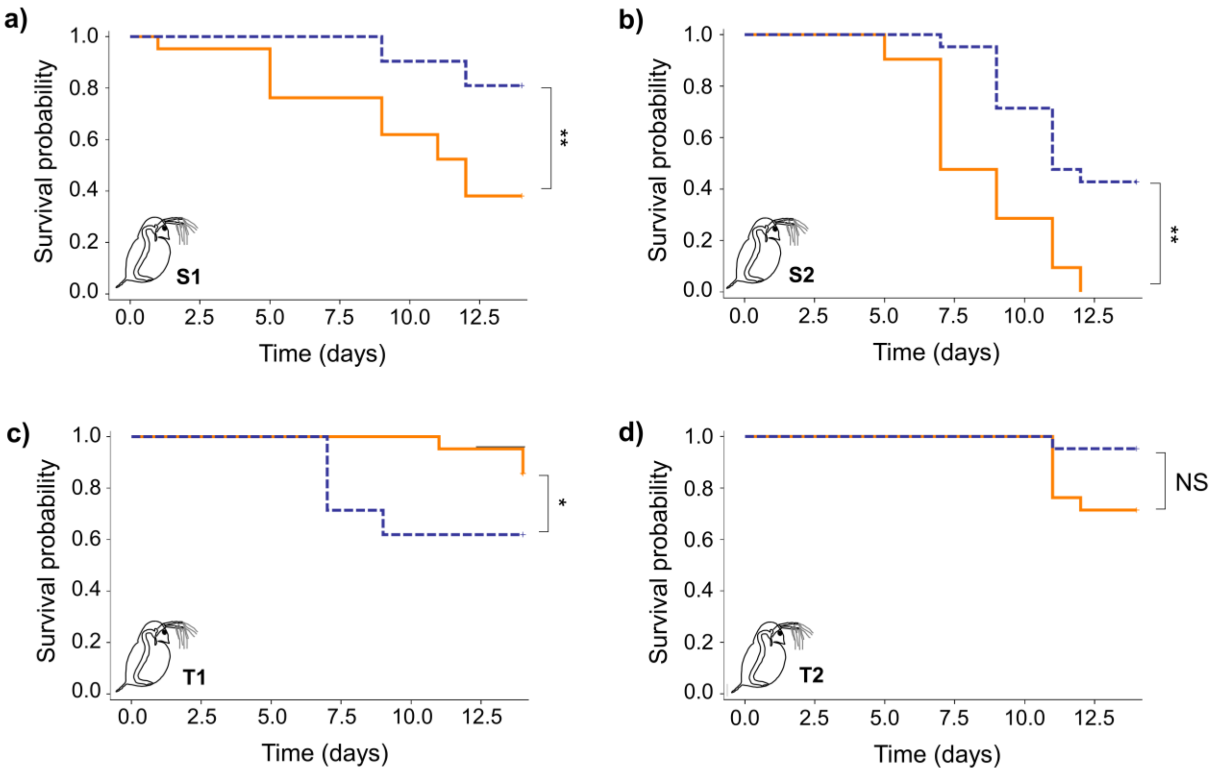

Supplementary Figure 1. Comparison of survival upon exposure to toxic cyanobacterial *versus* non-toxic green algal diet in the four experimental genotypes, prior to any disinfection or transplant treatment. a, b, Susceptible genotypes *S1* and *S2*, respectively, **c, d,** Tolerant genotypes *T1* and *T2*, respectively. Blue lines correspond to survival upon non-toxic green algal diet, and the orange lines to the toxic cyanobacterial diet. NS, not significant; *P<0.05; **P<0.01; ***P<0.0001 (pairwise comparisons in cox proportional hazard model). Sample size was n=21 (7 individuals * 3 replicates) for each genotype * diet combination.

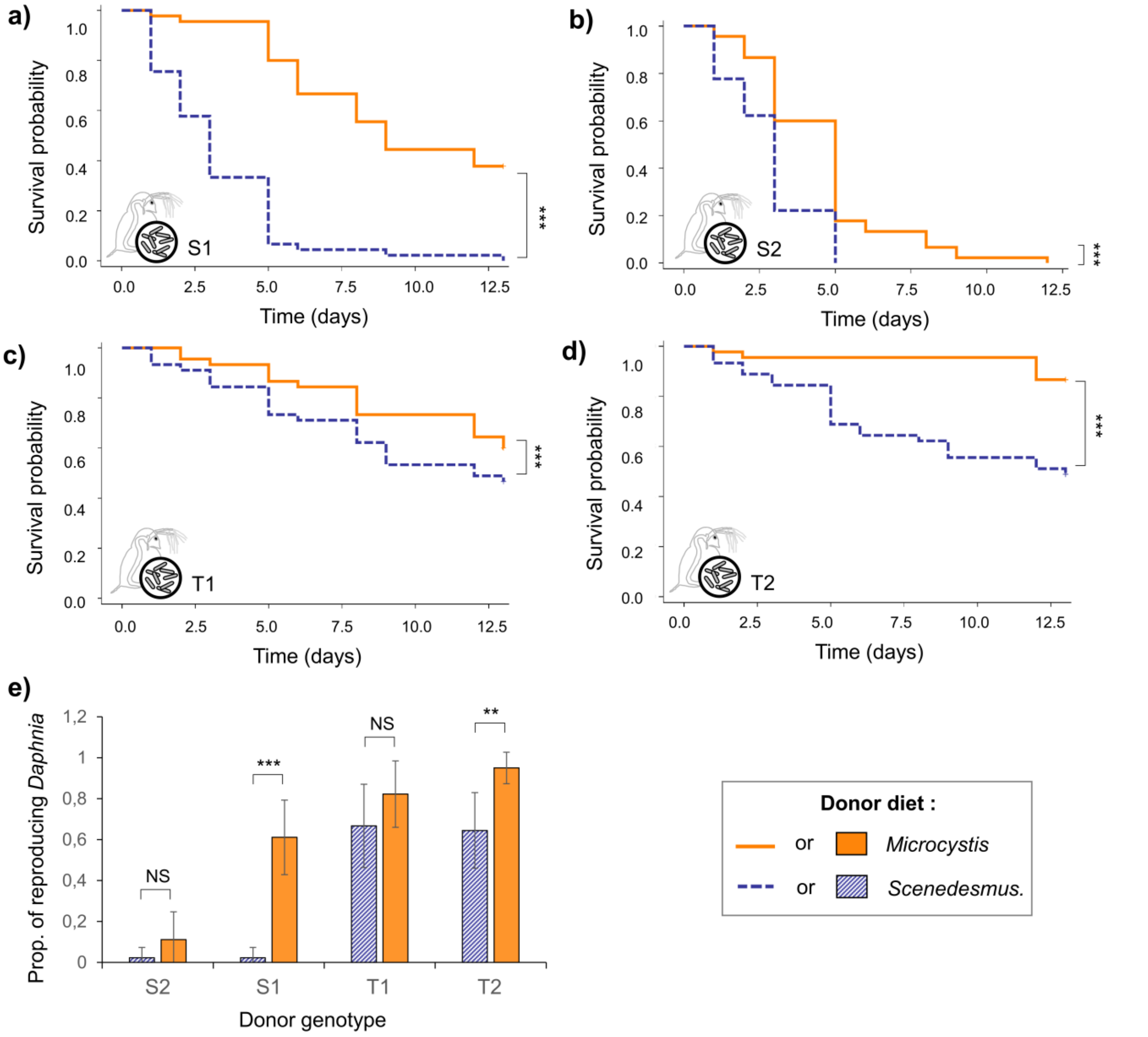

Supplementary Figure 2. Beneficial effects of a toxic cyanobacterial pre-exposure of donors vary between donor genotypes. a-d Effects of donor diet on survival, averaged over recipient genotypes, for *S1* (a), *S2* (b), *T1* (c) and *T2* (d) donor genotypes. The risk of mortality upon exposure to toxic cyanobacteria when the microbiota was preconditioned on a *Microcystis* compared to a *Scenedesmus* diet was reduced approximately 20 times in *S1* (a) and *T2* (d) donor genotypes (hazard ratio: 0.049 and 0.051, respectively), and approximately 5 times in *S2* (b) and *T1* (c) donor genotypes (hazard ratio: 0.21 and 0.18, respectively). **e,** Effects of donor diet on the probability of reproducing, averaged over recipient genotypes. NS, not significant; *P<0.05; **P<0.01; ***P<0.0001 (pairwise comparisons in cox proportional hazard model for survival, or in the logistic regression for reproduction). Error bars correspond to 95% confidence limits. The total sample size was n = 360 (4 donor genotypes * 2 donor diets * 3 recipient genotypes * 3 biological replicates * 5 individuals per replicate)

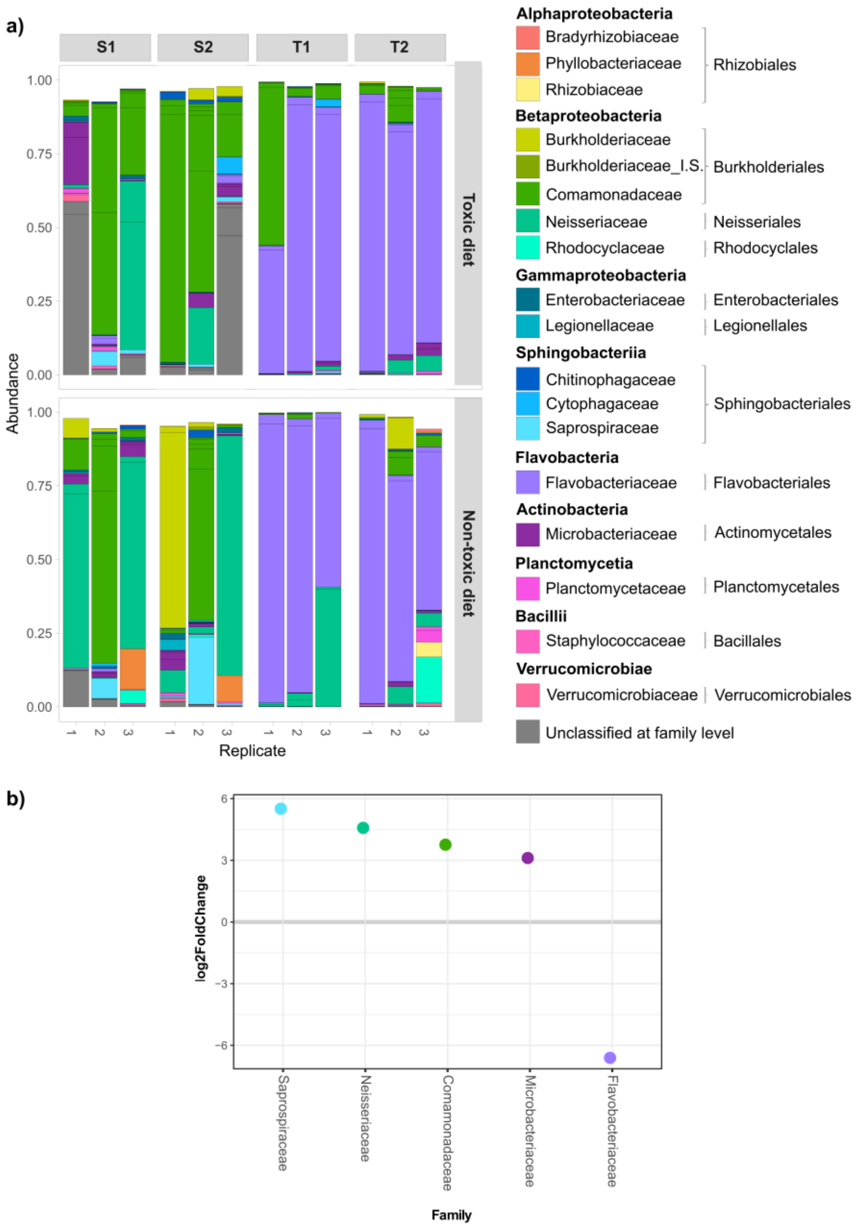

Supplementary Figure 3. Gut microbiota composition, at the family level, in the four *Daphnia* genotypes exposed to either a toxic cyanobacterial or a non-toxic green algal diet. a, Relative abundance of OTUs. Colors indicate bacterial families. *S1* and *S2* indicate susceptible genotypes, and *T1* and *T2* indicate tolerant genotypes (n = 3 replicated populations per type of genotype * diet combination). For each population, the microbiota characterization was performed on a pool of twenty guts. **b,** Gut bacterial families that exhibited significant differences between resistant and susceptible genotypes. The log2 fold change estimate was used to quantify the difference of family-level OTU abundance in susceptible versus tolerant genotypes. The circles indicate the ratio susceptible/tolerant.

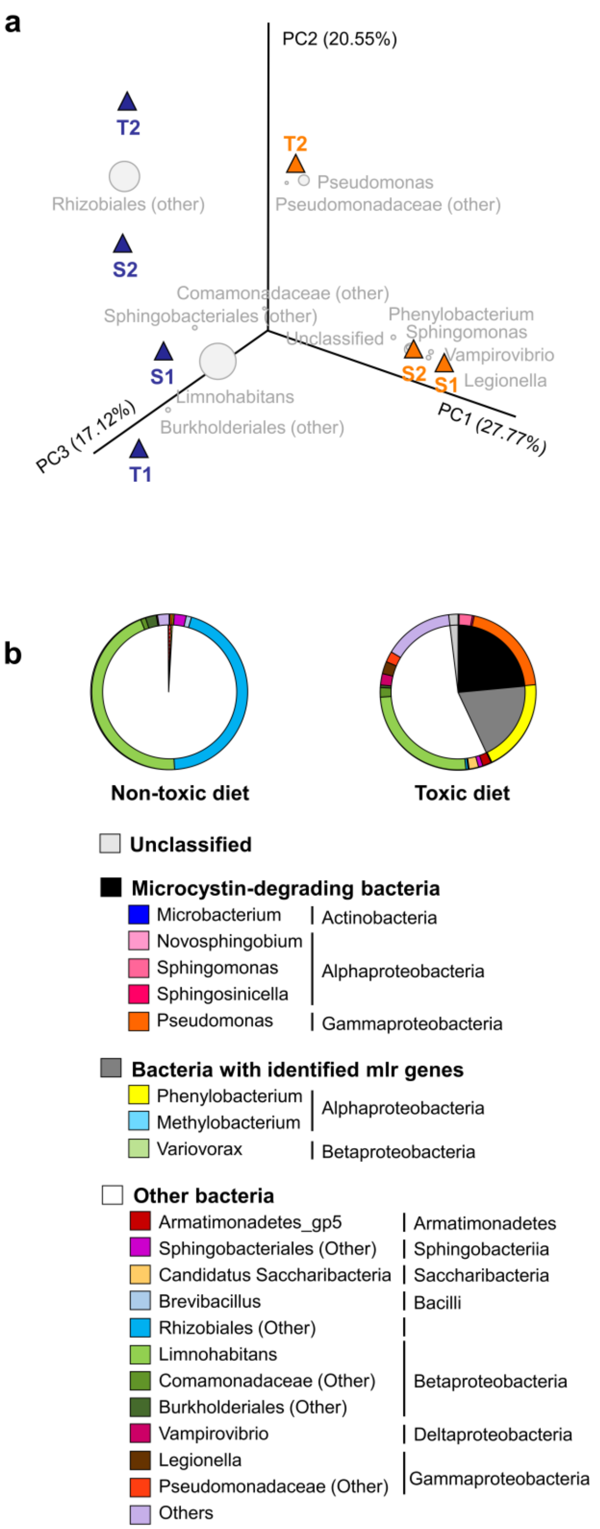

Supplementary Figure 4. Pilot test suggesting an alteration of the gut microbiota composition in response to cyanobacterial exposure. The gut microbial community of the two tolerant (*T1* and *T2*) and the two susceptible (*S1* and *S2*) genotypes exposed to green algal *versus* cyanobacterial diet (no data for genotype *T1* in the cyanobacterial diet) was characterized through 454 pyrosequencing on 16S rRNA (V3-V4 region), and data were analyzed with the QIIME software, as described in Callens et al.[1]. A single sample was characterized for each genotype * diet combination, so that the relative contribution of genotype and diet could not be tested statistically. **a,** Unweighted UniFrac Principal Coordinate Analysis of gut microbial communities in green algal (blue triangles) *versus* cyanobacterial (orange triangles) diet. The twelve more abundant bacterial taxa and groups are plotted as relative abundance-proportional grey circles. **b,** Potential microcystin-degradation activity in *Daphnia* gut microbiota, under green algal *versus* cyanobacterial diet. The outer circle indicates bacterial taxa, whereas the inner part of the pie-chart indicates the proportion of bacteria that have been identified as microcystin-degrading bacteria (black) and bacteria possessing genes involved in the microcystin degradation pathway (grey)[2,3].

Supplementary References

1. Callens, M. *et al.* Food availability affects the strength of mutualistic host–microbiota interactions in *Daphnia magna*. *ISME J.* **10,** 911–920 (2016).
2. Dziga, D., Wasylewski, M., Wladyka, B., Nybom, S. & Meriluoto, J. Microbial degradation of microcystins. *Chem. Res. Toxicol.* **26,** 841–852 (2013).
3. Mou, X. Z., Lu, X. X., Jacob, J., Sun, S. L. & Heath, R. Metagenomic identification of bacterioplankton taxa and pathways involved in microcystin degradation in Lake Erie. *PLoS One* **8,** 14 (2013).
